# Synthesis of Hydroxyapatite, β-Tricalcium Phosphate and Biphasic Calcium Phosphate Particles to Act as Local Delivery Carriers of Curcumin: Loading, Release and In Vitro Studies

**DOI:** 10.3390/ma11040595

**Published:** 2018-04-12

**Authors:** Despoina Xidaki, Panagiota Agrafioti, Dimitra Diomatari, Archontia Kaminari, Eleftherios Tsalavoutas-Psarras, Polyxeni Alexiou, Vasilios Psycharis, Effie C. Tsilibary, Spyridon Silvestros, Marina Sagnou

**Affiliations:** 1 STEP@biomaterials PC, Department of Research & Development, TEPA “Lefkippos”, Patriarchou Grigoriou & Neapoleos 27, 153 41 Agia Paraskevi, Athens, Greece; dx@step-biomaterials.gr (D.X.); lefti_7@yahoo.gr (E.T.-P.); etsilibari@gmail.com (E.C.T.); 2 Dental School, National and Kapodistrian University of Athens, Thivon 2, 115 27 Goudi, Athens, Greece; pennyagr@gmail.com (P.A.); irodiomatari@gmail.com (D.D.); silvestrosperio@gmail.com (S.S.); 3 Institute of Biosciences & Application, NCSR “Demokritos”, Patriarchou Grigoriou & Neapoleos 27, 153 41 Agia Paraskevi, Athens, Greece; kaminari@bio.demokritos.gr (A.K.); xenia.alexiou@gmail.com (P.A.); 4 Institute of Nanoscience & Nanotechnology, NCSR “Demokritos”, Patriarchou Grigoriou & Neapoleos 27, 153 41 Agia Paraskevi, Athens, Greece; v.psycharis@inn.demokritos.gr

**Keywords:** hydroxyapatite, β-tricalcium phosphate, biphasic calcium phosphates, curcumin, delivery

## Abstract

The successful synthesis of hydroxyapatite (HA), β-Tricalcium phosphate (β-TCP) and two biphasic mixtures (BCPs) of the two was performed by means of wet precipitation. The resulting crystals were characterized and the BCP composition was analyzed and identified as 13% HA—87% TCP and 41% HA—59% TCP. All samples were treated with curcumin solutions, and the degree of curcumin loading and release was found to be proportional to the TCP content of the ceramic. No further cytotoxicity was observed upon MG-63 treatment with the curcumin-loaded ceramics. Finally, the alkaline phosphatase activity of the cells was found to increase with increasing content of TCP, which provides an encouraging proof of concept for the use of curcumin-loaded synthetic biomaterials in bone remodeling.

## 1. Introduction

Healing of bone defects in adults closely resembles bone formation during organogenesis, and it is a complicated and multifactorial biological phenomenon. The whole process includes inflammatory responses, reactive oxygen species production, collagen production, mineralization, angiogenesis, osteoblast activation and osteoclast inhibition and it all occurs in the presence of a bone substitute scaffold. The main role of such a bone substitute would be to provide the necessary scaffold for all these biological events to occur in the right order, keeping the homeostasis of the area and occasionally contributing towards the successful completion of the process by its morphological and chemical attributes.

Calcium phosphate ceramics represent a category of bioceramics that have been widely used in the field of bone regeneration. Moreover, they have served as biological cements, coatings, drug delivery systems, and tissue engineering scaffolds. Hydroxyapatite (HA, Ca_10_(PO_4_)_6_(OH)_2_), β-tricalcium phosphate (β-TCP, Ca_3_(PO_4_)_2_), and biphasic calcium phosphates (BCP, mixtures of HA and β-TCP in a variety of ratios) [1,2,3], are the most commonly and successfully employed forms of calcium ceramics. They exhibit pronounced resemblance to bone tissue minerals, excellent biocompatibility, good cell attachment properties for ensuring natural biodegradability, bioresorption, and finally, they are easy and cost-effective to produce [4,5,6]. Over the years, a variety of factors that affect the composition and characteristics of the final products, including reaction pH, sintering temperature and duration, initial reagent purity and ratios, have been investigated. The resulting products have shown distinct chemical and physical characteristics, such as porosity, crystal size, grain size and roughness [7,8,9,10], with consequent effects on their functionality in both in vitro and in vivo models of bone regeneration [11,12,13].

The development of novel calcium phosphate ceramics as biomaterials has been primarily driven by the clinical need to further improve their properties and physicochemical characteristics for filling in osseous defects and voids. Moreover, they have also become promising candidates for novel local treatments able to address the increasing scientific and clinical interest. Calcium-phosphate crystals have attracted scientific attention as a drug delivery system aiming to deliver the desired drug concentration in a slow-release fashion whilst minimizing the effective dose of the drug, and hence the maximum plasma concentration and the potential side effects produced by systemic administration. These types of ceramics, and in particular HA, exhibit outstanding surface interaction properties and physicochemical characteristics, making them potential candidates for delivering steroids and hormones [14], antibiotics [15,16,17,18], statins as BMP-2 stimulants [19], vitamins [20] and anticancer drugs [21,22].

Turmeric, an Indian spice derived from the rhizomes of the plant *Curcuma longa*, has a long history of use in Ayurvedic medicine, due to the pharmacological action of its constituents including the three curcuminoids: curcumin (diferuloylmethane; the primary constituent and the one responsible for its vibrant yellow color), demethoxycurcumin, and bisdemethoxycurcumin, as well as volatile oils (tumerone, atlantone, and zingiberone), sugars, proteins, and resins [23]. Curcumin was first identified in 1910 by Lampe and Milobedzka [24], and since then, there has been an increasing number of literature reports and clinical trial protocols each year exploring the multifunctional biological and therapeutic potential of this molecule. Curcumin has been shown to regulate a number of biological factors and events that are crucial to inflammation, including transcription factors, cytokines, protein kinases, adhesion molecules, redox status and enzymes. Consequently, through moderation of the inflammation process, which plays a major role in most chronic illnesses, including neurodegenerative, cardiovascular, pulmonary, metabolic, autoimmune and neoplastic diseases, an outstanding therapeutic potential has been attributed to this natural product [25]. Dental applications of curcumin have also been investigated during the last decade [26]. In antimicrobial photodynamic therapy, curcumin has been extensively investigated as a photosensitizer in light-emitting diode (LED) activation techniques for periodontal and orthodontic cases [27,28,29,30,31]. Moreover, various formulations of curcumin have exhibited potential as adjunct therapies for professional scaling and root planning for periodontal disease treatment or general oral hygiene and mouthwash agents [32,33,34].

We would like to report herein the synthesis of hydroxyapatite (HA), β-tricalcium phosphate (β-TCP) and two biphasic calcium phosphates (BCPs) by the wet precipitation method and their infra-red, XRD and SEM characterization. The resulting materials were evaluated for their curcumin loading and release capacity, as well as for their cytotoxicity profile and alkaline phosphatase activation potential. To the best of our knowledge, this is the first time that the effect of curcumin uptake on synthetic bone substitutes has been investigated, aiming to reveal the potential application of synthetic bone substitutes as local curcumin delivery vehicles for bone healing applications.

## 2. Materials and Methods

### 2.1. Experimental

The identification of the crystal phases in the obtained products was carried out by the X-ray diffraction (XRD) technique, using the SIEMENS D500 diffractometer (Siemens AG, Karlsruhe, Germany) with a Cu–Kα radiation, a diffracted beam monochromator and the following combination of slits 1.0°/1.0°/1.0° as aperture diaphragms, 0.15° and 0.6° as detector and diffracted beam monochromator diaphragm respectively. The diffraction diagrams were recorded in the 2θ range 20–60° in 5 s per 0.03°, and they were analyzed with the Rietveld method. FT-IR was performed using a Perkin-Elmer spectrophotometer Spectrum RX I FT-IR (PerkinElmer, Boston, MA, USA). The KBr disk technique was used with 2 mg of powder in 200 mg of spectroscopic grade KBr (Merck KGaA, Darmstadt, Germany). Infrared spectra were recorded in the 4000–400 cm^−1^ region. The texture of the solids was examined by scanning electron microscopy (SEM) using a JEOLJSM-6300 instrument (JEOL USA, Inc.). Curcumin I was obtained by flash silica gel column chromatography of a sample of commercial curcumin (1 g) using chloroform followed by a chloroform:methanol mixtures of increasing polarity (100:0 to 95:5) as eluents. Sample purity was confirmed by means of nuclear magnetic resonance spectroscopy (NMR, Bruker Avance DRX 500 MHz) [35], infrared spectroscopy (IR, Perkin-Elmer spectrophotometer Spectrum RX I FT-IR, PerkinElmer, Boston, MA, USA) and mass spectroscopy Finnigan MAT TSQ 7000 (Finnigan MAT, San Jose, CA, USA). All necessary culture media and reagents were purchased from Biochrom, Berlin, Germany unless otherwise stated. For the MTT experiments absorbance was recorded by Sirio S Seac RADIM-Group Diachel ELISA plate reader (Radim Spa, Pomezia, Italy). The nitrogen adsorption/desorption isotherms at 77 K for surface area BET determination were measured in an automated volumetric system (AUTOSORB-1, Quantachrome Instruments, Boynton Beach, FL, USA). Prior to measurement, the samples were outgassed at 250 °C for 12 h.

### 2.2. Synthesis

#### 2.2.1. Method 1

A solution of (NH_4_)_2_HPO_4_ powder (10.3 g, 75.6 mmol) was dissolved in distilled water (330 mL) at 37 °C, under continuous stirring. Ca(NO_3_)_2_·4H_2_O powder (27.6 g, 167.4 mmol) was subsequently added to the above solution, followed by the addition of NH_4_OH (6.3 mL, 25 vol %) to the opaque solution almost immediately after the addition of calcium nitrate. The reaction mixture was stirred for 140 min at constant temperature. Precipitates were immediately separated from the container by filtration and dried at 60 °C for 24 h. Subsequent sintering at a temperature of 850 °C for 4 h resulted in the formation of Sample A. Addition of 200 mL NH_4_OH to the initial mixture and sintering of the final product at 1050 °C for 4 h, resulted in the formation of Sample B.

#### 2.2.2. Method 2

A solution of Ca(NO_3_)_2_·4H_2_O (10.2 g, 62 mmol) in distilled water (248 mL) was warmed to 40 °C and the pH adjusted to 10.5 by addition of NH_3_/H_2_O 2:1 solution (Solution A). Similarly, (NH_4_)_2_HPO_4_ (3.18 g, 23.5 mmol) was dissolved in distilled water (154 mL) and the resulting solution was warmed to 40 °C and the pH adjusted to 8 by addition of NH_3_/H_2_O (1:2) (Solution B).

Solution B was added dropwise to solution A, at a rate of 2 mL/min, while the temperature was raised to 65 °C. The final solution was stirred, under nitrogen, for 2 h. Then, it was cooled in ice water for an hour, filtered, and washed with ice water. The white precipitate formed was dried in an oven at 60–70 °C for 24 h. Finally, sintering at 950 °C for 4 h resulted in the formation of Sample C, whereas a 24 h aging (standing after precipitation) before oven-drying and sintering resulted in the formation of Sample D.

All samples were characterized by means of FT-IR, SEM and XRD, and their phase composition was determined.

### 2.3. Curcumin Loading

A solution of curcumin I in absolute ethanol was prepared (1 mg/mL). The synthetic ceramic substituent (<150 μm granule size, 200 mg) was suspended and incubated in the curcumin solution for 72 h at 18 °C under continuous stirring. Preliminarily, a qualitative kinetic study was performed, taking aliquots (500 μL) to measure the absorption at 425 nm, which is the λ_max_ wavelength for curcumin dissolved in ethanol, at time intervals of 48, 72, and 96 h and for each material (data not shown). There was no alteration of curcumin absorption between 72 and 96 h, hence the study was performed for 72 h of incubation. The suspension was then centrifuged at 4000 rpm for 20 min. The supernatant was collected and concentrated until dry under vacuum, and the resulting remaining curcumin I solid was redissolved in 4 mL ethanol. The absorbance at λ_max_ = 425 nm was recorded, and the amount of curcumin was determined based on the standard curve values of concentration against absorption. The percentage of loaded curcumin was calculated based on the initial curcumin concentration (1 mg/mL). All experiments were performed in triplicate.

### 2.4. Curcumin Release

A sample of curcumin loaded ceramic (100 mg) was suspended in Phosphate Buffered Saline (PBS, 2 mL). The suspension was incubated for 24 and 48 h at 37 °C, under continuous stirring. After 24 h, the suspension was centrifuged at 4000 rpm for 20 min, and aliquots from the supernatant (500 μL) were removed to be spectrophotometrically measured at 354 nm, which is the λ_max_ wavelength for curcumin dissolved in PBS/ethanol, and to determine the exact concentration of curcumin at this time point. The same procedure was also repeated for samples incubated for another 24 h, and, based on the standard absorption against concentration curve, curcumin concentration was determined, and hence the amount of curcumin released at this time point. The percentage of release at each time point was calculated based on the curcumin amount loaded per mg of material after the loading studies. All experiments were performed in triplicate.

### 2.5. Cell Cytotoxicity—MTT Assay

For cytotoxicity evaluation, the human osteoblast-like cell line MG-63 was used. Cells were grown in DMEM medium supplemented with 10% fetal bovine serum in the presence of 1% l-glutamine 200 mM and 1% penicillin/streptomycin in humidified 5% CO_2_ incubator at 37 °C. Cells were seeded in 96-well plates at a density of 2000 cells/well, and after they reached the desirable confluence (70–80%), the cells were starved for 24 h in DMEM medium with stable glutamine supplemented with 1% Fetal bovine serum (FBS) and 1% penicillin/streptomycin. Freshly prepared samples of HA, BCPs and β-TCP, with or without curcumin, were introduced to the cell cultures after they had been irradiated for 30 min under the UV light and kept in an ultrasonic bath to sustain homogeneity, at final concentrations of 200, 100, 50, 30, and 10 μg/mL, for 72 h. The supernatant was then removed and the cells were incubated in MTT solution (1 mg/mL) in DMEM for 4 h at 37 °C to form insoluble formazan crystals. The formazan crystals were dissolved in Dimethyl sulfoxide. The concentration of formazan crystals formed in the viable cells was estimated by measuring the absorbance at 570 nm.

### 2.6. Alkaline Phosphatase Activity Assay

The differentiation of treated MG-63 osteoblasts was determined by ALP activity assay. Cells were starved for 24 h and the next day they were treated with β-TCP, BCPs and HA with or without curcumin at 50 μg/mL concentrations in DMEM medium with 1% FBS for 5 days. The medium was removed, and the cells were thoroughly washed with 1× PBS. The cells were then incubated with 200 μL of *p*-Nitrophenyl phosphate substrate solution (Sigma-Aldrich, St. Louis, MO, USA) for 30 min in the dark. The ALP activity of the cells was determined by measurement of absorbance at 405 nm on a 96-well spectrophotometer.

Statistical Analysis. Data are presented as mean ± standard error of the mean (SEM). All experiments were performed in triplicate. Significance was defined as *p* < 0.05 by using a Students *t*-test.

## 3. Results and Discussion

### 3.1. Synthesis and Characterization

The synthesis of β-TCP was found to be a straightforward procedure, which consistently resulted in the desired product. The amount of ammonia added and the pH fluctuations were able to affect the purity of the β-TCP phase, whereas increasing temperature did not seem to alter phase composition dramatically. From the various experimental conditions attempted, it may be rationalized that the increase in the pH resulted in an increase in the HA percentage. This is in agreement with previously reported protocols [36,37], and may be related to the phase transformation of β-TCP to HA in alkaline environments. On the other hand, the formation of HA and the changes in phase composition giving rise to BCPs seem to have been affected by sintering temperature, as well as the re-dissolution period or aging allowed to the sample after the reaction time was complete.

**Figure 1:**
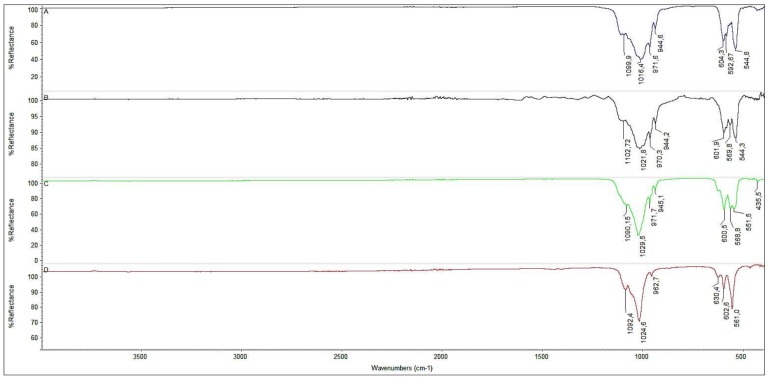
FT-IR spectra of 100% β-TCP (**A**), BCP ((**B**) 13% HA—87% TCP, (**C**) 41% HA—59% TCP) and 100% HA (**D**).

Figure 1 shows the characteristic FT-IR spectra of all samples. The characteristic peak at 3571 cm^−1^ was attributed to the stretching mode of hydroxyl group (OH^−^), whereas the bending mode resulted in a peak at 630 cm^−1^ peaks and both peaks strongly and clearly appear in HA samples, such as D. The peaks at the range of 557–604 and 945–1087 cm^−1^ may be assigned to the phosphate group (PO_4_^3−^).

Two sharp peaks at 604 and 544 cm^−1^ are characteristic of the pure β-TCP phase, and they clearly appear in Sample A, whereas the three characteristic peaks at 630, 600 and 560 cm^−1^ present in sample D are assigned to symmetrical and asymmetrical bending of the hydroxyapatitic phosphate group (PO_4_^3−^). BCPs are characterized by mixed and slightly shifted peaks at about 600, 570 and 540 cm^−1^ (Sample B and C). All the peak patterns in the FT-IR spectra of Samples A–D are in good agreement with related literature reports [38]. Moreover, the absence of any strong absorbance peak at 1400–1600 cm^−1^ and at about 875 cm^−1^ suggests that no carbonate group is present in any sample.

**Figure 2:**
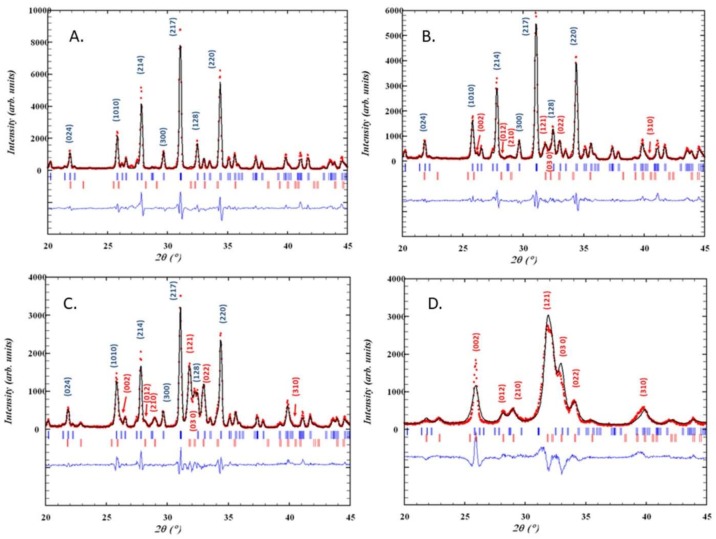
X-ray diffraction patterns of samples 100% β-TCP (**A**), BCP ((**B**) 13% HA—87% β-TCP, (**C**) 41% HA—59% β-TCP) and 100% HA (**D**) refined by the Rietveld method. The red points are the experimental points, the continuous black lines correspond to the calculated diagrams, blue and red vertical bars (|) at the bottom indicate the position of the Bragg peaks for the β-TCP and HA phases, respectively. The continuous line at the bottom is the difference between the experimental and theoretical intensity values. Blue Miller index values correspond to TCP and the red ones correspond to ΗΑ.

The qualitative XRD patterns, analyzed by the Rietveld method, for the four samples prepared are shown in Figure 2. All samples appeared to be highly crystalline, as indicated by the narrow and sharp peaks and the fairly even baseline. Three main peaks were observed for sample A at 2θ angle of 27.781, 31.062 and 34.451, which are in good agreement with the predicted main peaks of β-TCP, while sample D presented two main peaks at 31.974 and 33.093 and a smaller one at 28.341, which correspond well to the HA crystal structure. Samples B and C clearly show the presence of both phases in different proportions, and the percentage weight content for each phase was obtained by the Rietveld method and using the program FULLPROF [36]. The structural models used to identify and characterize the two structures have been previously reported [39,40]. The weight percent content for samples B and C were (13%:HA)/(87%:β-TCP) and (41%:HA)/(59%:β-TCP), respectively.

**Figure 3:**
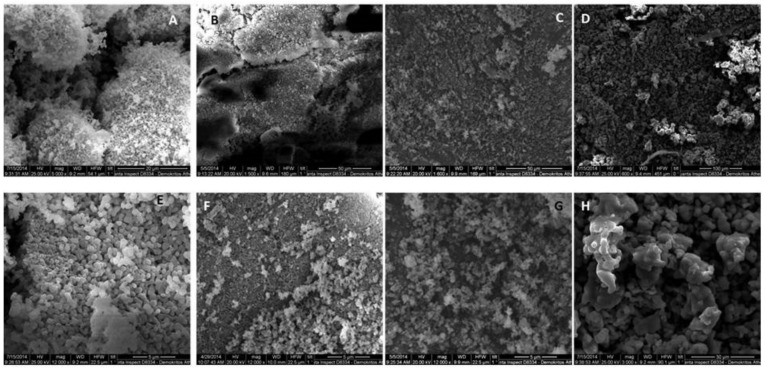
SEM photomicrographs of the 100% β-TCP (**A**,**E**), BCP ((**B**,**F**) 13% HA—87% β-TCP; (**C**,**G**) 41% HA—59% β-TCP) and 100% HA (**D**,**H**). (**A**–**D**) Low magnification, (**E**–**H**) high magnification.

SEM images of the prepared material are presented in Figure 3. At both magnifications, all surfaces appear to be rough and granular, with β-TCP showing the maximum effect followed by HA whereas BCP show less of this rough and uneven surface pattern. β-TCP exhibits both micro- and macropores, suggesting a pore network arrangement, which may be responsible for its increased solubility. Moreover, it is anticipated to allow better fluid uptake, cell accommodation and increased specific area for extensive physicochemical phenomena to take place. Increasing HA content results in the gradual decrease in surface roughness with anticipated consequences in solubility, cell anchoring and interactions, as well as fluid movements and exchanges. Similar findings have been reported before [41], although our preparation gave rise to enough roughness in the BCPs to anticipate a more favorable biological and physicochemical behavior compared to the literature preparations.

### 3.2. Curcumin Loading and Release

**Figure 4:**
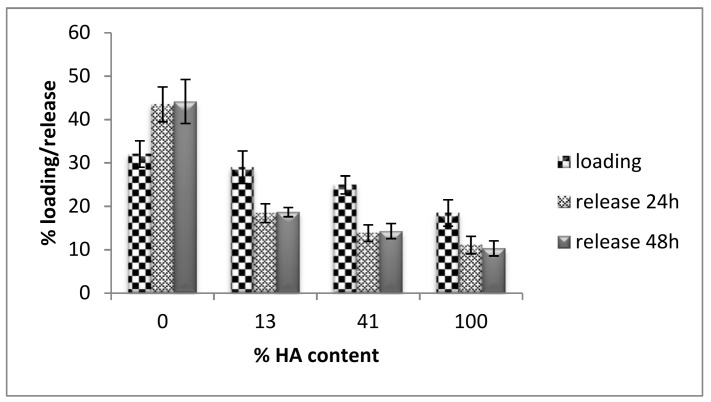
The percentage loading and release of curcumin on samples of 100% β-TCP, BCP (13% HA–87% β-TCP, 41% HA–59% β-TCP) and 100% HA. The results are mean ± standard deviation (SD) of three independent experiments. Loading was performed in ethanol solution for 72 h at 18 °C. Release studies were performed in PBS solution for 24 h and 48 h.

The anticipated differences in physicochemical properties of the four samples presumably related to the crystal structure and porosity are truly confirmed by the results of curcumin loading and release to the various ceramics. More specifically, the results depicted in Figure 4 show that the maximum curcumin uptake is exhibited by β-TCP, and there is a decreasing trend to the loading capacity as the HA component increases. In a similar manner, curcumin release follows the same trend, revealing that β-TCP not only adsorbs the maximum quantity of curcumin, but is also able to unload it and release it effectively. On the contrary, HA shows equally limited ability to either uptake or release the curcumin molecules, and finally, the gradual increase of HA phase in the BCPs results in a gradual decrease in both curcumin loading and release.

In the case of curcumin loading, bearing in mind that an ethanolic solution of curcumin was used to incubate the ceramics, the more hydrophobic HA would have been expected to interact better with the curcumin solution. Still, the fact that HA exhibited the smallest loading may imply that surface area is a more determining factor affecting the uptake, since β-TCP would present a more extended area to interact with the curcumin solution. This suggestion seems to be in good agreement with our Specific Surface Area measurements BET (SSA) of the four ceramics, as they are presented in Table 1, where increasing the TCP ratio resulted in an increase in SSA. Our results, therefore, indicate that the existence of macro-, meso-, or microporosity, and the corresponding surface area, seems to affect the degree of curcumin release as well. Previous investigations [42,43] have also concluded that specific area and pore dimensions play a crucial role in the loading and release of bioactive molecules on potential drug delivery systems such as porous HA/collagen microspheres and Si-containing bioactive glass [42,43]. HA and β-TCP have similar chemical structures, but they differ in their crystalline structure, which in its turn causes differences in their solubility. The order of curcumin release seems to follow the trend of solubility and Ca^2+^ release from phosphate bioceramics, i.e., β-TCP > BCPs > HA [44]. The β-diketone moiety of curcumin has affinity and interacts with Ca^2+^ atoms following, therefore, the fate of calcium from the crystal structure into solution.

### 3.3. Cell Cytotoxicity

**Figure 5:**
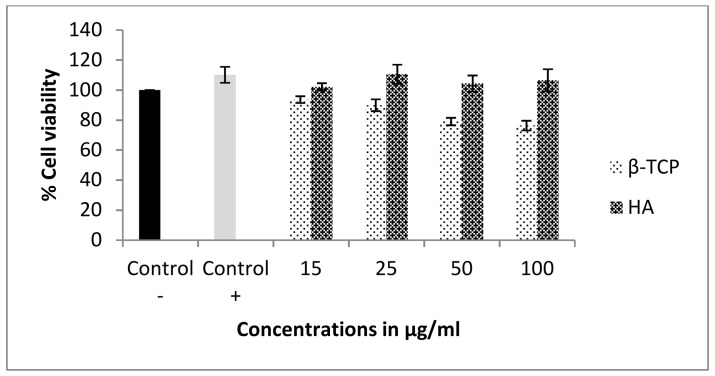
The effect of β-TCP and HA on MG-63 cell viability. Cells were treated with 15, 25, 50 and 100 μg/mL of each bone graft. Two control groups were used, control-maintained at 1% FBS and control + maintained at 10% FBS. The results are mean ± standard error (SE) of three independent experiments.

**Figure 6:**
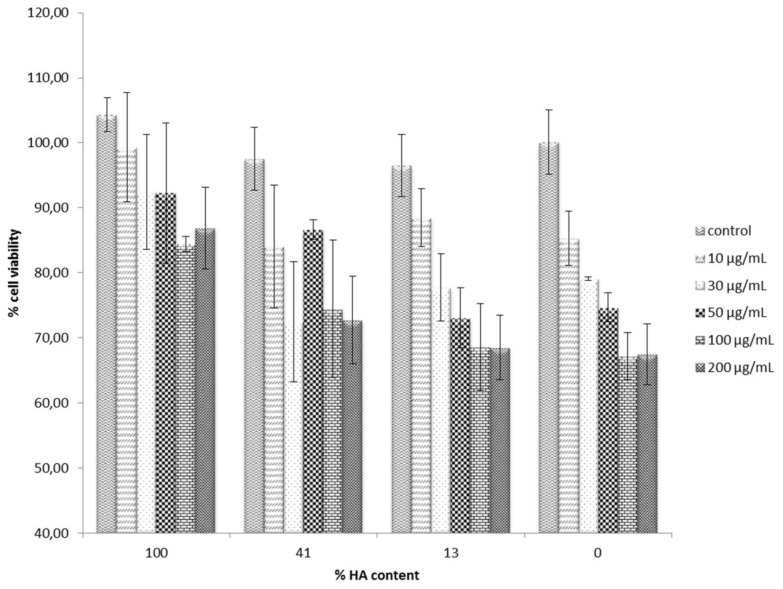
The effect of curcumin-loaded β-TCP, HA and biphasic samples on MG-63 cell viability and proliferation. Cells were treated with 10, 30, 50, 100 and 200 μg/mL of each bone graft. The results are presented as mean ± standard deviation (SD) of three independent experiments.

The MG-63 cells were used to evaluate the potential cytotoxic effect of either the synthesized calcium ceramics alone or after they had been immersed in curcumin solution and curcumin had been adsorbed. Initially, the individual cytotoxicity of β-TCP and HA apatite alone was evaluated, and the results are shown in Figure 5. Cells were treated with either form of calcium phosphate for 3 days, and MTT (3-(4,5-Dimethylthiazol-2-yl)-2,5-diphenyltetrazolium bromide) colorimetric analysis was used to measure the viability of cells, which interacted with different concentrations of them. Cell viability was expressed as a percentage of the untreated control. In the case of β-TCP osteoblasts, viability decreased in a dose-dependent manner with the increasing concentration of the ceramic. However, even at the highest concentration tested, 100 μg/mL, only a slight cell survival decrease was observed, rendering the material non-toxic at this concentration range. On the other hand, increasing concentration of HA did not seem to affect cell growth and proliferation at all under these experimental conditions. These results are in good agreement with previous studies, which explored the biological effect of various forms of HA on MG-63 cells, focusing on the size, shape and dimensions of the particles [44,45]. It was, therefore, of great significance to investigate whether curcumin adsorption would result in any significant increase in toxicity. The role of curcumin in bone metabolism has not yet been studied in great detail, and is even controversial. Some antiosteoclastogenic properties of curcumin have been reported, which implies a regulatory role of curcumin in bone resorption, whereas under other experimental conditions and systems, high curcumin concentration markedly inhibited the proliferation of rat calvarial osteoblastic cells and induced the death of cancerous human osteoblast [46]. Figure 6 presents the results of the cell cytotoxicity evaluation of the curcumin-bearing products. In all cases, the lowest cell survival was recorded at the highest ceramic concentration, and there was a dose—dependent cell toxicity increase. It is, however, noteworthy that in all cases of curcumin-loaded HA, the effect of curcumin in cell cytotoxicity was minimal, since it was only reduced from 100% to about 87% at the highest concentration of 200 μg/mL. In contrast, more dramatic changes in cell survival were observed upon increasing the concentration of both curcumin-loaded BCPs and β-TCP.

In a qualitative approach, the dose-response of increasing the concentration of curcumin-loaded calcium phosphates appears to follow the order of β-TCP > BCPs > HA. It may, therefore, be suggested that it follows the order of the increase in curcumin release, β-TCP > BCPs > HA, as has been previously evaluated. Although curcumin is a safe and well-tolerated, clinically used natural product, there is plenty of literature evidence that it can exhibit cell cytotoxicity against certain tumor cells and even act as a chemotherapeutic agent with multi-potent mode of action. Still, at the concentrations of 100 μg/mL in β-TCP, which was found to both adsorb and release the most curcumin molecules, cell survival was only reduced to 67–70%, compared to an 80% cell survival when no curcumin was loaded on the ceramic. Overall, these results indicate a safe in vitro profile, until at least 50 μg/mL graft concentration, and addition of this localized curcumin delivery did not cause any significant cell cytotoxicity increase to counterbalance any anticipated beneficial effect resulting from curcumin loading to the bone substitute. However, the effect of curcumin on osteoblast differentiation is still unknown.

### 3.4. Alkaline Phosphatase Activity Assay

**Figure 7:**
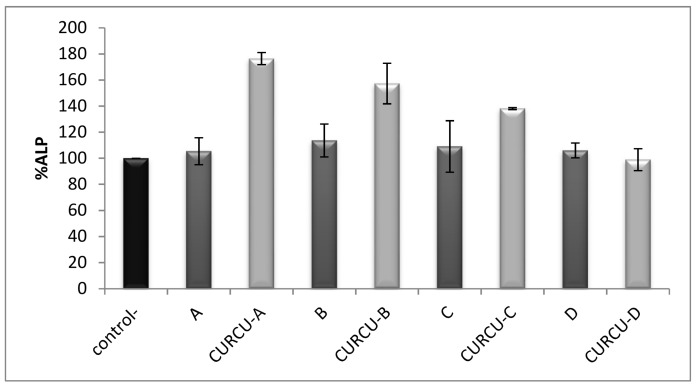
Quantification of ALP activity on MG63 cells 5 days after treatment with 50 μg/mL of each bone graft with or without curcumin loading. In the control group, control-, the cells were maintained in 1% FBS. The results are presented as mean ± standard error (SE) of three independent experiments. 100% β-TCP (**A**), BCP ((**B**) 13% HA—87% β-TCP, (**C**) 41% HA—59% β-TCP) and 100% HA (**D**).

Alkaline phosphate (ALP), is a marker of the osteoblastic phenotype and it is expressed when progenitor cells differentiate into osteoblasts. Therefore, any increase in the number of osteoblasts is anticipated to result in proportional ALP activity increases. The ALP assay is often used to evaluate cell differentiation abilities. More important is that it is generally accepted as a suitable osteoinduction marker. Figure 7 shows the results for the evaluation of ALP activity on MG63 cells 5 days after treatment with 50 μg/mL concentration of each bone graft with or without curcumin loading, compared to the control sample. In all cases of β-TCP, BCPs and HA, no significant differences in ALP activity were observed, and they did not seem to affect MG - 63 differentiation compared to the control. On the contrary, the ALP activity of osteoblast-like cells was found significantly increased by the curcumin treated samples of β-TCP and BCPs, whereas in the case of curcumin-loaded HA, there was still no significant effect on ALP activity compared to the control. It is noteworthy that the order of ALP activity increase follows the order of curcumin loading and release results.

Combining these two sets of results may allow us to suggest that the excessive loading and release capacity of β-TCP for curcumin observed, compared to rest of the ceramic bone substitutes, can cause this beneficial increase in cell differentiation. Previous studies have shown similar results for hydroxyapatitic particles and nanoparticles alone, since they did not affect in any significant way the cell differentiation of MG—63 as shown by ALP activity measurements [45]. Moreover, experiments with curcumin alone have shown that it can promote osteogenic differentiation of rat mesenchymal stem cells (rMSCs) and increased ALP activity and osteoblast-specific mRNA expression of Runx2 and osteocalcin [46]. Consequently, the increased ALP activity observed in our study may be attributed to the action of curcumin released from the ceramic upon incubation of MG—63 cells with the curcumin-loaded ceramics.

## 4. Conclusions

In this study, β-TCP, two BCPs and HA particles were successfully synthesized by the wet precipitation method and characterized by means of FT-IR, SEM and XRD to determine the phase composition and crystallinity of the products prepared. They were all incubated with curcumin, and β-TCP particles were found able to adsorb and release the highest amount of curcumin, followed by BCPs, whereas HA exhibited limited loading and release capacity. Cell cytotoxicity of the calcium phosphate ceramics was not significant in MG-63 osteoblast-like cell line. Even more encouraging was that upon curcumin loading, no dramatic increase of cell cytotoxicity was observed. Moreover, ALP activity increased significantly in the case of curcumin-bearing β-TCP samples, indicating that such a combination is beneficial in enhancing osteoblast differentiation. The results of our study demonstrate for the first time, to the best of our knowledge, that appropriate loading of curcumin on bone substitute materials of synthetic or natural origin and other related biomaterials used in bone healing and regeneration, can improve the osteoinductive characteristics of the substitute and may be of great benefit and clinical importance. Local release of the multimodal curcumin at the site of bone healing may indeed directly interfere with the cellular processes involved in bone regeneration and remodeling, regulating inflammation, angiogenesis cell proliferation and differentiation anticipating an overall improved clinical outcome.

## Figures and Tables

**Table 1:** Specific surface area of HA, β-TCP and biphasic calcium phosphate powders.

Sample (% HA)	Specific Surface Area BET (m^2^/g)
0	16.779
13	12.547
41	9.742
100	5.077
